# External assessment of an artificial intelligence-enabled electrocardiogram for aortic stenosis detection

**DOI:** 10.1093/ehjdh/ztaf067

**Published:** 2025-07-01

**Authors:** Darae Kim, Eunjung Lee, Jihoon Kim, Eun Kyoung Kim, Sung-A Chang, Sung-Ji Park, Jin-Oh Choi, Young Keun On, Zachi Attia, Paul Friedman, Kyoung-Min Park, Jae K Oh

**Affiliations:** Division of Cardiology, Department of Internal Medicine, Heart Vascular Stroke Institute, Samsung Medical Center, Sungkyunkwan University School of Medicine, Seoul, Republic of Korea; Department of Cardiovascular Medicine, Mayo Clinic, Rochester, MN, USA; Division of Cardiology, Department of Internal Medicine, Heart Vascular Stroke Institute, Samsung Medical Center, Sungkyunkwan University School of Medicine, Seoul, Republic of Korea; Division of Cardiology, Department of Internal Medicine, Heart Vascular Stroke Institute, Samsung Medical Center, Sungkyunkwan University School of Medicine, Seoul, Republic of Korea; Division of Cardiology, Department of Internal Medicine, Heart Vascular Stroke Institute, Samsung Medical Center, Sungkyunkwan University School of Medicine, Seoul, Republic of Korea; Division of Cardiology, Department of Internal Medicine, Heart Vascular Stroke Institute, Samsung Medical Center, Sungkyunkwan University School of Medicine, Seoul, Republic of Korea; Division of Cardiology, Department of Internal Medicine, Heart Vascular Stroke Institute, Samsung Medical Center, Sungkyunkwan University School of Medicine, Seoul, Republic of Korea; Division of Cardiology, Department of Internal Medicine, Heart Vascular Stroke Institute, Samsung Medical Center, Sungkyunkwan University School of Medicine, Seoul, Republic of Korea; Department of Cardiovascular Medicine, Mayo Clinic, Rochester, MN, USA; Department of Cardiovascular Medicine, Mayo Clinic, Rochester, MN, USA; Division of Cardiology, Department of Internal Medicine, Heart Vascular Stroke Institute, Samsung Medical Center, Sungkyunkwan University School of Medicine, Seoul, Republic of Korea; Department of Cardiovascular Medicine, Mayo Clinic, Rochester, MN, USA

**Keywords:** Artificial intelligence, Electrocardiogram, Aortic stenosis

## Abstract

**Aims:**

To assess the performance of an artificial intelligence-enabled electrocardiogram (AI-ECG) algorithm in identifying patients with moderate to severe aortic stenosis (AS) in an Asian cohort from a tertiary care centre.

**Methods and results:**

We identified a randomly selected patients ≥60 years old who underwent echocardiography and ECG within in 31 days between 2012 and 2021 at the Samsung Medical Center in Korea. Patients with previous cardiac surgery, prosthetic valves, or pacemakers were excluded. The AI-ECG model, originally developed and validated by Mayo Clinic in the USA, was applied without fine-tuning. Performance metrics, including the area under the curve (AUC), sensitivity, specificity, positive predictive value (PPV), negative predictive value (NPV), and accuracy, were calculated to compare AI-ECG predictions with TTE-confirmed AS status. Among 5425 patients, 1095 had moderate to severe AS, and 4330 age- and sex-matched patients without AS were included as controls. The AI-ECG model achieved an AUC of 0.85 (95% CI: 0.84–0.87) in detecting moderate to severe AS. Sensitivity, specificity, PPV, NPV, and accuracy were 0.83, 0.65, 0.37, 0.94, and 68.29%, respectively. The model's performance was consistent across various age and sex subgroups, with sensitivity increasing in older patients.

**Conclusion:**

The AI-ECG model developed in the USA demonstrated comparable performance in detecting moderate to severe AS in an Asian cohort compared with its original validation population. These findings highlight the potential utility of AI-ECG as a non-invasive screening tool for AS across diverse patient populations.

## Graphical Abstract

**Graphical Abstract:**
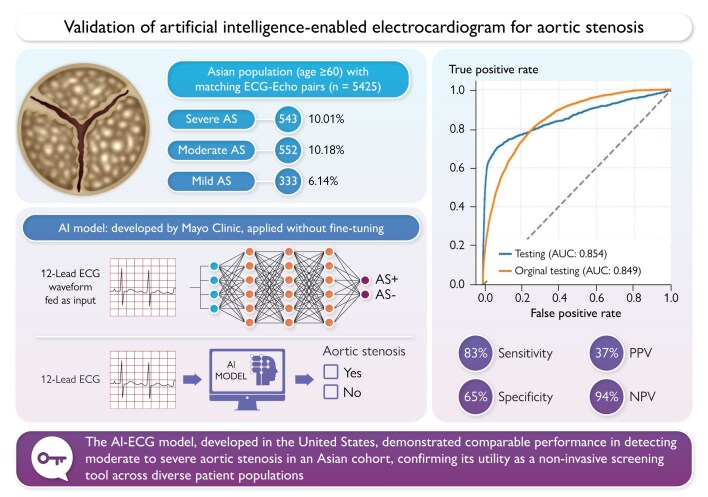

## Introduction

Artificial intelligence-enabled electrocardiogram (AI-ECG) has shown the potential for detecting various cardiovascular diseases. Recent large data set studies reported diagnostic accuracy of AI-ECG for detecting silent atrial fibrillation, left ventricular dysfunction, and determination of a person’s age and sex.^1–5^ Advanced AI methods facilitated detection of signals and patterns of ECG, which were largely unrecognized by human interpreters and have transformed ECG to a powerful, non-invasive tool to detect cardiovascular disease.

A recent paper by Cohen-Shelly *et al*.^6^ and Oh *et al*. demonstrated AI-ECG using a convolutional neural network to screen moderate to severe aortic stenosis (AS) patients. Screening for moderate to severe AS is important because asymptomatic severe AS will benefit from early surgery and there is potential for early intervention to delay progression.^7^ This model was well designed with high area under curved (AUC) of 0.85. However, although this model was derived from three geographically different centres in USA, majority of patients were Caucasian (88%) and generalizability of this model needs to be determined. In addition, the model has limitations in its diagnostic spectrum. Notably, the sensitivity, while reasonable at 75%, leaves room for improvement in terms of detecting a broader range of AS severity. Moreover, false-negative rates are a concern, with 17% of severe AS and 32% of moderate AS cases misclassified, which poses challenges in a screening context where early identification is critical for timely intervention. These limitations necessitate further exploration to ensure the model’s reliability in diverse clinical settings and populations. Therefore, in this study, we aimed to validate the performance of AI-ECG developed by the Mayo group in Asian population to determine the universal applicability of AI-ECG regardless of race.

## Patients and methods

### Study population

We identified 5425 patients with age ≥60 years who had at least one transthoracic echocardiography (TTE) and ECG performed within 31 days at Samsung Medical Center in Korea between January 2012 and December 2021. In this patients, we included those with one of following AS measurements: aortic valve area (AVA), mean pressure gradient (MPG), peak transaortic velocity, or dimensionless velocity index (DVI).^6,8,9^ Philips ECG machines with a 500 Hz sampling rate were used in the current study, differing from the GE machines in the original study, although both employed the same 500 Hz sampling rate. Patients with incomplete TTE parameters or incomplete or corrupted ECG waveforms were excluded. Patients with previous cardiac surgery, a prosthetic valve or pacemaker were also excluded. The final cohort was assigned for validation. The Institutional Review Board of Samsung Medical Center approved this study.

### Data sources and labelling

Based on the previous study,^6^ patients were categorized into two groups using TTE data: echo-positive AS (+) for those with moderate to severe AS and echo-negative AS (−) for those with mild or no AS, following established guideline.^9,10^

### Transthoracic echocardiogram

All patients underwent comprehensive 2D transthoracic echocardiography according to the current guideline.^9,11^ Peak velocity was measured by continuous-wave Doppler from several acoustic windows to obtain the highest maximal velocities. Peak pressure gradients across the AV were calculated using the simplified Bernoulli equation. The position that yielded the highest AV velocity was used, and at least three signals were traced and averaged to determine the time–velocity integral and to calculate the MPG.^12^ AVA calculated by the continuity equation and indexed for body surface area. Left ventricular outflow tract velocity was measured by pulsed wave Doppler. Patients were classified as having moderate to severe AS if they satisfied at least one of the following echocardiographic criteria: peak velocity ≥ 3.0 m/s, MPG ≥ 20 mmHg, DVI ≤ 0.35, or AVA ≤ 1.5 cm².

### Electrocardiogram

All ECGs were acquired as digital standard 12-lead 10-second ECGs using a Marquette ECG machine (GE Healthcare, WI, USA). Their raw data were stored using the MUSE data management system for later retrieval.

### Artificial intelligence model evaluation

**Figure 1:**
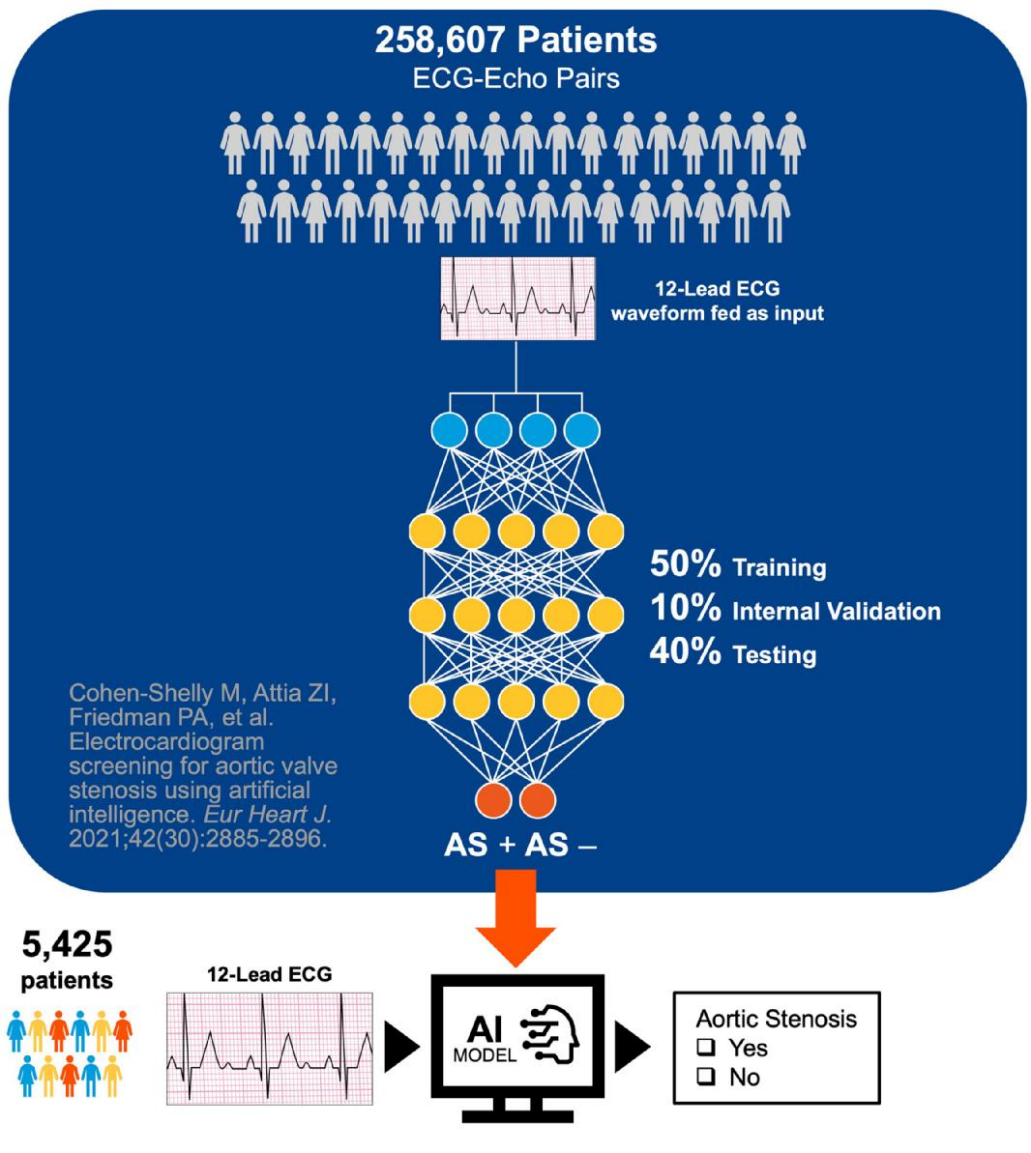
External performance assessment flow diagram.

The convolutional neural network developed for screening AS^6^ with 155 681 patients’ standard 12-lead ECGs was used for our cohort. No additional training was implemented. Overall flow is described in *Figure 1*.

### Statistical analysis

The receiver operating characteristic (ROC) curve was formulated to assess the previously developed model's ability to predict AI-ECG-positive AS compared with TTE-positive AS with our dataset. We applied the same threshold that was established a threshold during the validation phase when the AI-ECG was developed. We subsequently calculated standard diagnostic performance metrics, which include area under the curve (AUC), sensitivity, specificity, positive predictive value (PPV), negative predictive value (NPV), and accuracy.

Continuous variables were described as mean and standard deviation or median [inter-quartile range (IQR)] when appropriate. Categorical variables were presented as *n* (%). Statistical analyses were computed using Python scikit-learn library.

## Result

### Baseline and clinical characteristics

During study periods, 1095 patients with more than mild AS had valid ECG–TTE pairs. We identified age, sex-matched (4:1) patients without AS who had valid ECGs (*n* = 4330). Mean age was 74.56 ± 7.77 years with 2556 (47.12%) women. Median time interval between ECG and TTE was 0 days (IQR 0, 4) for moderate to severe AS patients. The prevalence of moderate to severe AS (TTE-positive AS) was 20.18% which is about five times compared with that of the training cohort for AI-ECG. Baseline clinical characteristics and AS severity distribution is described in *Table 1*. In this cohort, 10.01% (*n* = 543) had severe AS, 10.18% (*n* = 552) had moderate AS, and 6.14% (*n* = 333) had mild AS. Among severe AS, 24 patients (4.42%) were classified as low-flow, low-gradient (LF-LG) severe AS. All patients were Asian. Incidences of comorbidities including hypertension, diabetes mellitus, coronary artery disease, and chronic obstructive pulmonary disease (COPD) were significantly higher in AS (+) group compared with AS (−) group (*P* < 0.001). Clinical characteristics, echocardiographic data, and ECG parameters were compared across the four groups (*Table 2*). AVA, peak velocity, and MPG between true positive (TP) and false negative (FN) were significantly different.

**Table 1:** Patients’ characteristics and comorbidities

	*N* = 5425
Age, years	74.56 ± 7.77
Age group	
60–69	1502 (27.69%)
70–79	2444 (45.05%)
≥ 80	1479 (27.26%)
Female	2556 (47.16%)
AS severity	
No AS	3997 (73.68%)
Mild AS	333 (6.14%)
Moderate AS	552 (10.18%)
Severe AS	543 (10.01%)
Hypertension	985 (18.16%)
Myocardial infarction	233 (4.29%)
COPD	108 (1.99%)
Diabetes Mellitus	627 (11.56%)
CAOD	529 (9.75%)

AS, aortic stenosis; CAOD, coronary artery disease; COPD, chronic obstructive pulmonary Disease.

**Table 2:** Comparisons of clinical characteristics, echocardiography, and electrocardiogram parameters among four AI-enabled groups

	True positive	True negative	False positive	False negative	*P*
	(*n* = 905)	(*n* = 2800)	(*n* = 1530)	(*n* = 190)	
Age, mean ± SD	74.63 ± 7.82	74.73 ± 7.78	74.33 ± 7.73	73.65 ± 7.51	0.11
Female, *n* (%)	419 (46.3)	1146 (40.93)	901 (58.89)	90 (47.37)	<0.001
Hypertension, *n* (%)	669 (73.92)	72 (2.57)	116 (7.58)	128 (67.37)	<0.001
CAOD, *n* (%)	377 (41.66)	32 (1.14)	54 (3.53)	66 (34.74)	<0.001
COPD, *n* (%)	72 (7.96)	11 (0.39)	9 (0.59)	16 (8.42)	<0.001
Myocardial Infarction, *n* (%)	36 (3.98)	138 (4.93)	55 (3.59)	4 (2.116)	0.07
Diabetes mellitus, *n* (%)	323 (35.69)	138 (4.93)	96 (6.27)	70 (36.84)	<0.001
Echocardiography					
Aortic valve area, cm^2^	0.92 ± 0.29 (missing 10)	1.34 ± 0.23 (missing 2704)	1.35 ± 0.23 (missing 1383)	1.09 ± 0.23 (missing 6)	<0.001
Peak velocity, m/s	4.35 ± 0.94 (missing 2)	2.98 ± 0.33 (missing 2704)	2.95 ± 0.31 (missing 1379)	3.6 ± 0.57 (missing 4)	<0.001
Mean pressure gradient, mmHg	46.64 ± 22.29 (missing 2)	18.88 ± 4.4 (missing 2704)	18.89 ± 4.09 (missing 1379)	29.97 ± 10.28 (missing 4)	<0.001
LVEF, %	64.13 ± 7.76	NA	NA	60.34 ± 10.57	<0.001
ECG measurement (II lead)					
QRS duration, ms	98.61 ± 20.19	96.69 ± 19.09	91.35 ± 16.36	93.02 ± 14.84	<0.001
QT interval, ms	413.21 ± 37.61	408.44 ± 34.55	404.67 ± 32.5	406.73 ± 30.45	<0.001
QTc, ms	448.13 ± 31.94	439.79 ± 31.68	439.03 ± 27.17	440.28 ± 29.58	<0.001
P axis	44.3 ± 27.02	45.83 ± 25.75	43.74 ± 26.78	45.49 ± 26.86	0.06
R axis	26.93 ± 39.44	27.16 ± 42.6	28.55 ± 37.65	33.74 ± 39.03	0.06
T axis	80.64 ± 70.06	51.32 ± 45.58	43.92 ± 38.46	55.68 ± 51.11	<0.001
Heart rate, b.p.m.	71.8 ± 11.88	70.56 ± 10.81	71.66 ± 10.77	71.43 ± 11.46	<0.001

### External performance outcomes

**Figure 2:**
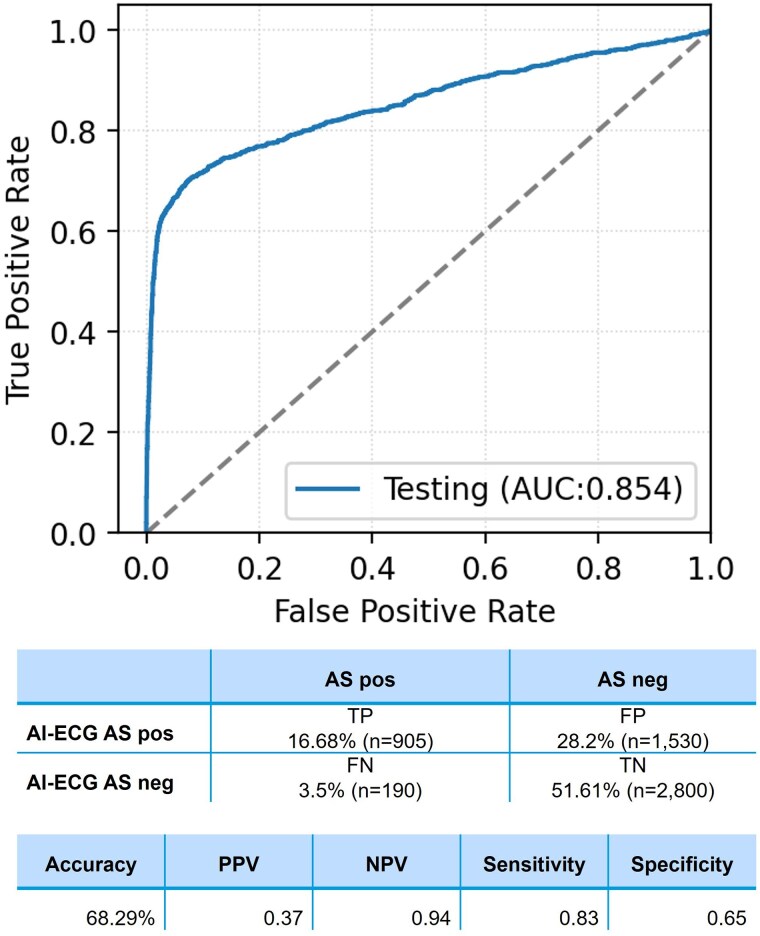
The receiver operating characteristic (ROC) curve of the convolutional neural network for identifying patients with moderate to severe aortic stenosis (AS).

Among 5425 patients, AUC of ROC was 0.85 (95% CI: 0.84–0.87). Sensitivity, specificity, PPV, NPV, and accuracy were 0.83, 0.65, 0.37, 0.94, and 68.29%, respectively. True positive was present in 16.68% (*n* = 905), true negative in 51.61% (*n* = 2800), false positive in 28.2% (*n* = 1530), and FN in 3.5% (*n* = 190) (*Figure 2*). Among the FNs, 83.68% had moderate AS, whereas only 11.57% of the false positives had mild AS (*Table 3*). Patients with true positive results more frequently exhibited comorbidities such as hypertension, COPD, coronary artery obstructive disease (CAOD), and diabetes compared with the other groups (*P* < 0.001) (*Table 4*). The false positive group showed a significantly higher prevalence of hypertension and CAOD and a higher proportion of females compared with the true negative group. Notably, ECG features, including QRS duration, QT interval, T-wave axis, and heart rate, differed significantly between the four groups (*P* < 0.001 for all comparisons).

**Table 3:** AI-enabled electrocardiogram model performances according to severity of AS

Testing group (*n* = 5425)	Aortic stenosis severity			
	Normal	Mild	Moderate	Severe
True positive (*n* = 905, 16.68%)	0 (0)	0 (0)	393 (43.43)	512 (56.57)
True negative (*n* = 2,800, 51.61%)	2644 (94.43)	156 (5.57)	0 (0)	0 (0)
False positive (*n* = 1,530, 28.2%)	1353 (88.43)	177 (11.57)	0 (0)	0 (0)
False negative (*n* = 190, 3.5%)	0 (0)	0 (0)	159 (83.68)	31 (16.32)

**Table 4:** Comparison of comorbidities between AS (+) and AS (−) groups

	Moderate to severe AS (−) (*n* = 4330)	Moderate to severe AS (+) (*n* = 1095)	*P*-value
Hypertension (%)	188 (4.34%)	797 (72.79%)	<0.001
Myocardial infarction (%)	193 (4.46%)	40 (3.65%)	0.28
COPD (%)	20 (0.46%)	88 (8.04%)	<0.001
Coronary artery disease (%)	86 (1.99%)	443 (40.46%)	<0.001
Diabetic mellitus (%)	234 (5.4%)	393 (35.89%)	<0.001

### Performances of AI-ECG models across age and sex subsets

**Figure 3:**
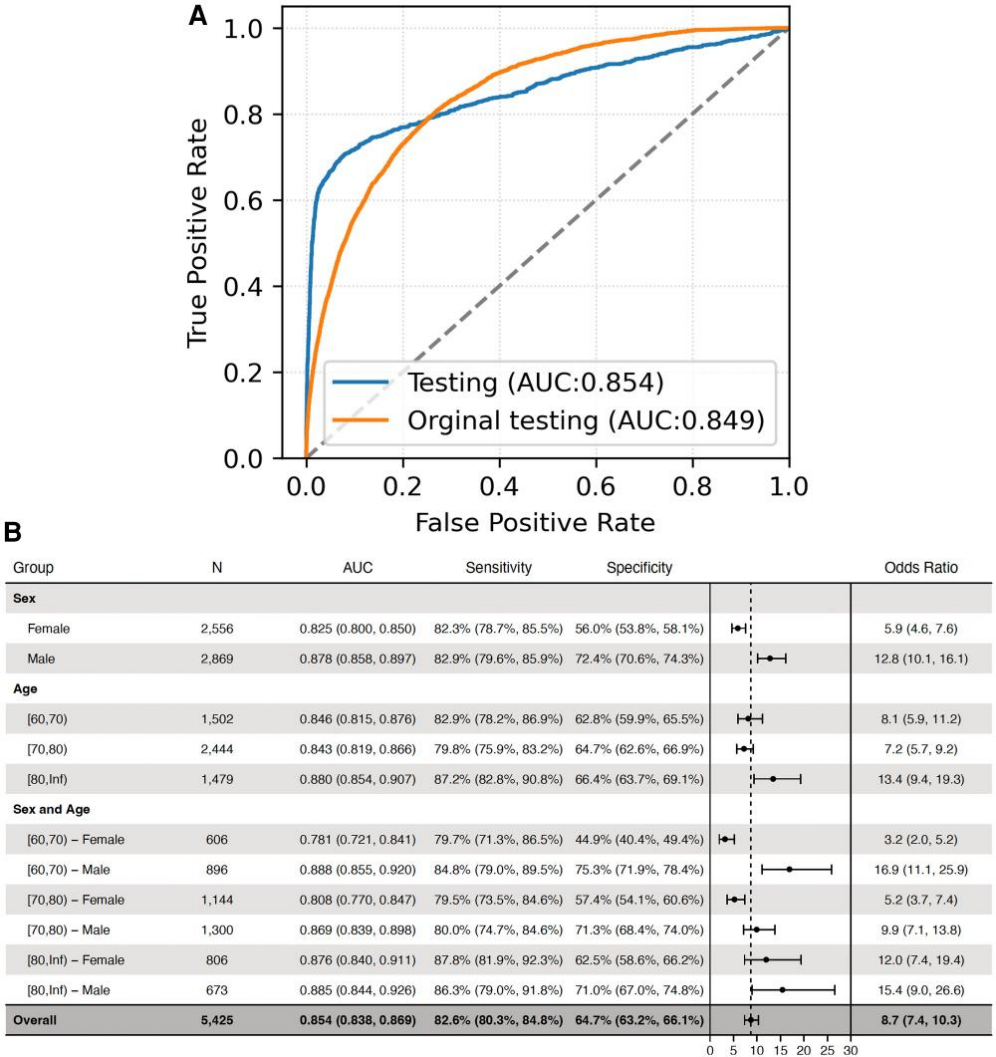
Receiver operating characteristic curves, sensitivity, and specificity across age and sex subsets. (*A*) The receiver operating characteristic curve of the convolutional neural network for identifying patients with moderate to severe aortic stenosis is shown for the Samsung Medical Center cohort (blue) and original testing cohort (orange). The area under the curve (AUC) is calculated. (*B*) The sensitivity and specificity for the detection of moderate to severe aortic stenosis labelled by artificial intelligence electrocardiogram are tabulated across a range of sex and age combinations for testing dataset. The diagnostic odds ratio (OR) and 95% confidence interval (CI) are shown.

**Figure 4:**
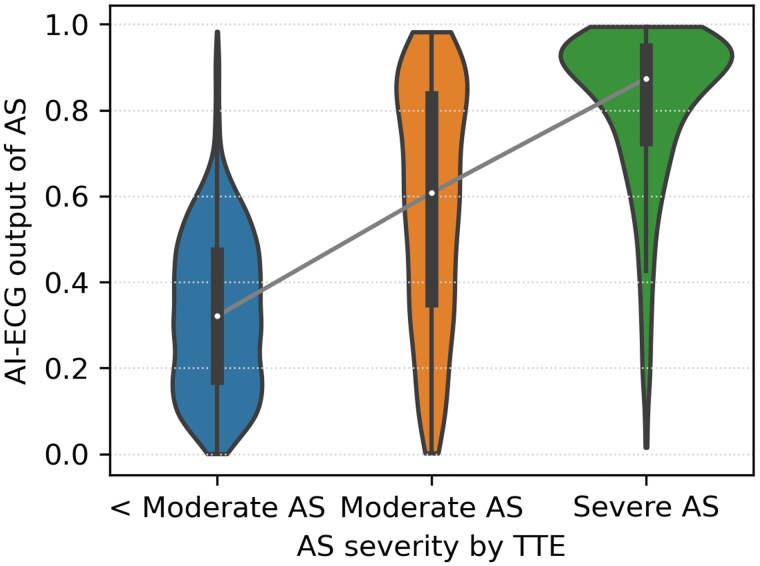
The output of aortic stenosis positive labelled by artificial intelligence electrocardiogram (AI-ECG) compared with transthoracic echocardiography (TTE) severity of aortic stenosis.

Analysis stratified by age and sex (*Figure 3B*) revealed that sensitivity and specificity progressively increased with advancing age. Women exhibited lower specificity compared with men across all age groups. Positive correlation was observed between AI-ECG and echocardiographic assessment of AS severity (*Figure 4*).

Kaplan–Meier survival analysis was conducted to explore the prognostic implications of AI-ECG classification and AS severity (see Supplementary material online, *Figure S1*). Survival curves revealed an unexpected trend where patients classified as severe AS exhibited better survival than those with moderate AS (see Supplementary material online, *Figure S1A*). Furthermore, when stratified by AI-ECG classification (TP, TN, FP, FN), FN patients exhibited the worst survival rates (see Supplementary material online, *Figure S1B*).

## Discussion

The AI-ECG model developed by the Mayo Clinic has demonstrated a robust ability to identify patients with moderate to severe AS. In our study, we aimed to externally assess its diagnostic performance in an Asian population, a demographic notably under-represented in the original development cohort. In this Asian cohort, the AI-ECG previously developed in Mayo clinic successfully identified patients with moderate to severe AS with high performance (AUC 0.85), despite different ethnic population and different prevalence of moderate to severe AS compared with that of Mayo (20.2 vs. 3.7%). The clinical significance of this external performance assessment lies in its confirmation that AI-ECG can reliably detect AS in populations with different ethnicity and baseline characteristics. This is particularly meaningful as it supports the broader applicability of the AI-ECG.

While the AUC was consistent between our study and the original cohort, differences in sensitivity (83 vs. 78%) and specificity (65 vs. 74%) likely reflect variations in the underlying patient populations. Our cohort, comprising older Asian patients from a tertiary care centre with a higher prevalence of moderate to severe AS (20.2 vs. 3.7%), inherently posed different challenges for the AI-ECG model. The higher sensitivity observed in our cohort may be attributable to the increased prevalence of disease-related comorbidities, such as hypertension and left ventricular hypertrophy, which might amplify ECG signal changes indicative of AS. Conversely, the lower specificity may result from the higher prevalence of overlapping conditions with similar ECG patterns.

The implications of the PPV and NPV are particularly important in the context of screening. In our study, the PPV was 37%, reflecting the higher prevalence of AS in our cohort, while the NPV remained high at 94%, underscoring the utility of the AI-ECG as a reliable exclusionary tool. However, the selection of a 4:1 control:AS population in this study resulted in an artificially elevated prevalence of moderate to severe AS (20%), which differs from the true prevalence in an unselected population. While this design enables a balanced evaluation of the AI-ECG model's diagnostic performance, it also impacts the test characteristics, such as PPV and NPV. Specifically, the PPV is higher and the NPV is lower compared with what would be observed in a population with a lower prevalence of AS. This highlights the importance of interpreting these metrics in the context of the study cohort. Despite these considerations, the high NPV suggests that the AI-ECG can effectively rule out moderate to severe AS in most cases, making it a valuable initial screening tool. By reliably identifying patients who do not require further evaluation, the AI-ECG has the potential to reduce the burden of unnecessary echocardiograms in low-risk populations. However, a positive AI-ECG result should always be confirmed through echocardiography before making clinical decisions.

The AI-ECG model demonstrated robust performance in detecting severe AS, however, its sensitivity in identifying moderate AS cases was relatively lower, as reflected by the higher proportion of FN patients with moderate AS (83.68%) compared with severe AS (16.32%). Subgroup analyses of moderate AS group revealed that FN cases within moderate AS group generally have less severe hemodynamic profiles compared with TP cases within moderate AS group (see Supplementary material online, *Table S1*). Although FN cases within moderate AS group showed significantly lower peak AV velocity and MPG compared with TP cases within moderate AS group, the difference was small. The subtle differences in echocardiographic parameters may have contributed to the model’s FNs.

The diagnostic spectrum of the model also highlights certain limitations. While the sensitivity of 75% is acceptable, it indicates a gap in detecting a full spectrum of AS severity. Of particular concern are the false-negative rates: 17% for severe AS and 32% for moderate AS. These findings raise challenges in screening applications, where timely and accurate detection is critical for early intervention and improved outcomes. FNs in severe AS could delay necessary treatments like valve replacement, while undetected moderate AS cases may progress to more severe disease stages, potentially leading to adverse outcomes. These limitations highlight the need for cautious clinical application and potential refinement of the model. Despite these limitations, the model may be capturing fundamental physiological markers of AS rather than population-specific characteristics. The ability of the AI-ECG to generalize across diverse cohorts enhances its utility as a non-invasive, cost-effective screening tool in varied clinical settings, potentially improving early diagnosis and management of AS on a global scale.^13^

Since this study primarily focuses on the diagnostic performance of AI-ECG, survival analysis was conducted as a supplementary exploratory analysis rather than a key study endpoint. Kaplan–Meier curves (see Supplementary material online, *Figure S1*) revealed that patients classified as severe AS had better survival outcomes than those with moderate AS. This paradoxical result is likely explained by higher rates of timely aortic valve interventions in severe AS patients, which have well-documented survival benefits. Additionally, the retrospective nature of our study and the use of all-cause mortality as an endpoint may have introduced confounding factors, including non-cardiovascular causes of death. Comparison of prognosis of TP, TN, FP, and FN showed that TP and TN had similar survival patterns, which may suggest that AI-based classification does not directly correlate with long-term prognosis. FN had the worst prognosis and were predominantly patients with a high burden of comorbidities. These findings highlight an important limitation of the AI-ECG model in risk stratification, as it may fail to identify the highest-risk patients. However, it is important to note that this study was not designed to evaluate the prognostic implications of AI-ECG, and these results should be interpreted with caution. While AI-ECG demonstrated strong diagnostic performance in detecting AS, these findings suggest that it should not yet be considered a reliable tool for prognostic stratification or risk assessment. Future research should incorporate cardiovascular-specific mortality indicators, multivariable adjustments for clinical factors and interventions, and prospective study designs to further explore whether AI-ECG can be refined to enhance risk stratification beyond disease detection.

AS represents a critical valvular heart disease that significantly impacts patient outcomes, particularly as the disease progresses to moderate or severe stages.^14–17^ Early detection of AS is paramount as it allows for timely intervention, which can significantly improve the prognosis, particularly in asymptomatic patients.^7^ This is crucial because the natural history of untreated severe AS often leads to adverse outcomes, including heart failure and sudden death.^18–20^ Therefore, accurate and early prediction tools are essential in the clinical management of AS, offering an opportunity to intervene before the onset of irreversible cardiac damage.

Recent advances of AI in healthcare have allowed for significant strides in the automated detection of cardiovascular diseases using ECG.^2,5,21,22^ Most AI-ECG models such as the model we validated in this paper capitalize on large-scale datasets and sophisticated neural network architectures, which have been crucial in learning complex and nuanced patterns within ECG signals. This approach goes beyond traditional diagnostic methods, offering an objective and consistent means of detecting conditions that might otherwise be overlooked by human interpreters. The strength of these models lies in their ability to handle vast amounts of data and detect subtle, often imperceptible changes associated with disease states, making AI-ECG a powerful tool in the early identification and management of AS.

Importantly, our findings align with the broader trend in healthcare AI, where deep learning models are increasingly validated across diverse populations, enhancing their generalizability and clinical applicability. The AI-ECG’s ability to maintain high accuracy across different ethnic groups suggests that the underlying features identified by deep learning are not solely dependent on population-specific characteristics but are instead capturing universal physiological signals related to AS. This highlights the robustness of AI models in transcending traditional clinical boundaries and adapting to varying patient demographics. Moreover, our work underscores the potential for AI-ECG to provide continuous, real-time monitoring capabilities that could complement existing clinical workflows. By integrating these AI-driven tools into routine practice, clinicians can achieve a more pro-active approach to managing AS, particularly in at-risk populations where early detection is critical.

### Study limitation

Several limitations of our study should be acknowledged. Our external performance assessment was conducted in a single centre with a cohort entirely composed of Asian patients. Although this allows for assessing the model’s generalizability to an ethnic group under-represented in the original model, it may not fully capture the variability seen in other populations or different clinical settings. Further multicentre validation across diverse demographic groups is necessary to strengthen the evidence for broader clinical application.

The AI-ECG model used was trained on a dataset with a lower prevalence of moderate to severe AS compared with our cohort. This discrepancy could impact the model’s performance, particularly in populations with a higher prevalence of disease, potentially altering sensitivity and specificity metrics. The prevalence of moderate to severe AS in our cohort (20%) was higher than that of unselected populations due to the use of a 4:1 control:AS ratio. This design affects certain test characteristics, particularly PPV and NPV, and limits the generalizability of these findings to broader populations. Future studies should evaluate the AI-ECG model in unselected populations to better understand its real-world screening performance. Additionally, our study population was restricted to individuals aged 60 years and older. This age threshold was chosen because AS is more prevalent in older populations, with a significantly higher incidence in individuals above 60 years. The prevalence of moderate to severe AS in the general population ranges from 2% to 4% but increases markedly with age, reaching ∼5% to 10% in those over 60 years.^6^ While the focus on older patients may appear to limit the generalizability of our findings to younger populations, we believe that focusing on individual above 60 year old age group does not introduce substantial selection bias but rather aligns with the clinical need for accurate AS detection in populations most at risk.

We used an age- and sex-matched control group rather than the entire population to provide a balanced comparison for diagnostic performance metrics, in line with the original AI-ECG study. While this selection approach led to a higher prevalence of moderate to severe AS (20.2%) compared with the general population, it enabled a more focused evaluation of the model's performance.

Also, in this study, among FN patients, 83.68% were classified as moderate AS, while only 16.32% were classified as severe AS. This suggests that FN cases primarily resemble moderate AS, indicating potential limitations of the AI-ECG model in detecting subtle hemodynamic features associated with moderate AS. Addressing these challenges may improve the model's sensitivity in this subgroup. While the AI-ECG model demonstrated strong diagnostic performance, its ability to accurately stratify long-term clinical risk remains uncertain. The observed limitations in prognostic differentiation, particularly among high-risk patients, underscore the need for further refinement. These findings emphasize that AI-ECG may serve as a useful screening tool but should not yet be considered a validated risk stratification method for AS outcomes. Future research should focus on integrating prognostic factors.

Moreover, our analysis did not involve re-training or fine-tuning the AI-ECG model on the Asian cohort data. While this preserves the integrity of the original model's performance assessment, it may also limit the potential to enhance its diagnostic accuracy further when applied to this specific population. Future studies could explore fine-tuning techniques to optimize the model for varying clinical environments and patient characteristics.

Lastly, the model's interpretability remains a challenge. Although deep learning models excel at pattern recognition, the ‘black-box’ nature of these algorithms limits our ability to fully understand the decision-making process, potentially complicating clinical acceptance. Efforts to develop more interpretable AI models, which can provide insights into the critical ECG features influencing predictions, would be valuable in bridging the gap between AI-ECG outputs and clinical reasoning.

## Conclusion

In this study, we ‘conducted an external performance evaluation’ of an AI-ECG model originally developed in a predominantly Caucasian population at Mayo Clinic for detecting moderate to severe AS in an independent Asian cohort. The AI-ECG demonstrated robust performance, with an AUC of 0.85, comparable to results from the original cohort, despite notable differences in ethnic composition and disease prevalence. These findings support the generalizability of the AI-ECG model across diverse populations and highlight its potential as a valuable, non-invasive screening tool for AS in clinical settings.

## Supplementary Material

ztaf067_Supplementary_Data

## Data Availability

The AI-ECG AS algorithm used in this study was developed by Mayo Clinic. External validation using the AI-ECG AS model is available upon request (jae.oh@mayo.edu). However, the dataset used in this study is not publicly available due to restrictions outlined in our institutional review board approval.
