# Supplementary material for: External assessment of an artificial intelligence-enabled electrocardiogram for aortic stenosis detection
Source: Eur Heart J Digit Health. 2025 Jul 1;6(4):656–64. doi: 10.1093/ehjdh/ztaf067 (PMC12282354; doi:10.1093/ehjdh/ztaf067)
Supplement: ztaf067_Supplementary_Data [file ztaf067_supplementary_data.zip › Supplementary table 1.pdf]

Supplementary Table 1. Moderate AS subgroup analysis

	True positive	False negative	P
	(n = 393)	(n = 159)	
Aortic valve area, cm^2	1.10 ± 0.26	1.12±0.21	0.435
Peak velocity, m/s	3.64±0.38	3.50±0.30	<0.001
Mean pressure gradient, mmHg	29.61±5.59	27.69±5.14	<0.001
Dimensionless velocity index	0.31±0.07	0.32±0.07	0.406
