# Supplementary figures and images for: External assessment of an artificial intelligence-enabled electrocardiogram for aortic stenosis detection
Source: Eur Heart J Digit Health. 2025 Jul 1;6(4):656–64. doi: 10.1093/ehjdh/ztaf067 (PMC12282354; doi:10.1093/ehjdh/ztaf067)

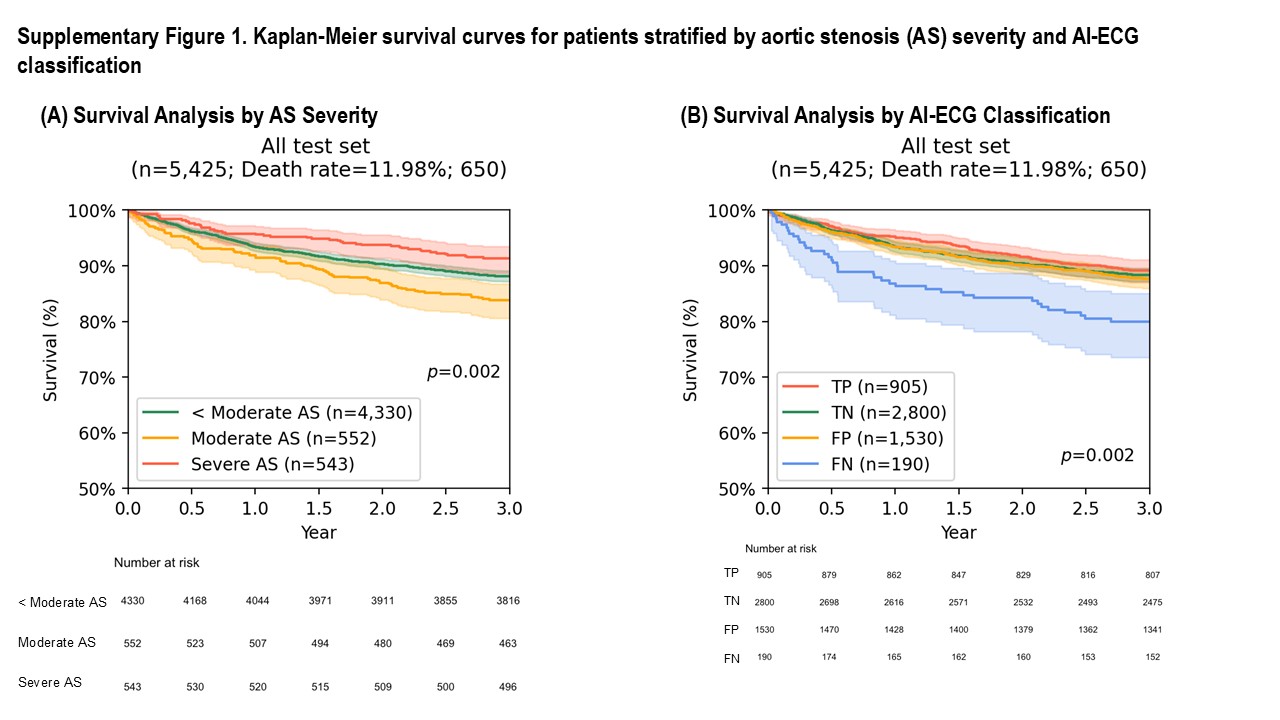

Supplement: ztaf067_Supplementary_Data [file ztaf067_supplementary_data.zip › SupF (1).jpg]
